# Rapid Detection of *Salmonella enterica* in Food Using a Compact Disc-Shaped Device

**DOI:** 10.3390/mi7010010

**Published:** 2016-01-15

**Authors:** Shunsuke Furutani, Mitsutoshi Kajiya, Narumi Aramaki, Izumi Kubo

**Affiliations:** 1 Health Research Institute, National Institute of Advanced Industrial Science and Technology (AIST), 1-8-31 Midorigaoka, Ikeda, Osaka 563-8577, Japan; shunsuke-furutani@aist.go.jp; 2 Graduate School of Engineering, Soka University, 1-236 Tangi, Hachioji, Tokyo 192-8577, Japan; e1056103@soka.ac.jp (M.K.); e15m5601@soka-u.jp (N.A.)

**Keywords:** PCR, rapid detection, *Salmonella enterica*, compact disc, food, meat

## Abstract

Rapid detection of food-borne pathogens is essential to public health and the food industry. Although the conventional culture method is highly sensitive, it takes at least a few days to detect food-borne pathogens. Even though polymerase chain reaction (PCR) can detect food-borne pathogens in a few hours, it is more expensive and unsatisfactorily sensitive relative to the culture method. We have developed a method to rapidly detect *Salmonella enterica* by using a compact disc (CD)-shaped device that can reduce reagent consumption in conventional PCR. The detection method, which combines culture and PCR, is more rapid than the conventional culture method and is more sensitive and cheaper than PCR. In this study, we also examined a sample preparation method that involved collecting bacterial cells from food. The bacteria collected from chicken meat spiked with *S. enterica* were mixed with PCR reagents, and PCR was performed on the device. At a low concentration of *S. enterica*, the collected *S. enterica* was cultured before PCR for sensitive detection. After cultivation for 4 h, *S. enterica* at 1.7 × 10^4^ colony-forming units (CFUs)·g^−1^ was detected within 8 h, which included the time needed for sample preparation and detection. Furthermore, the detection of 30 CFUs·g^−1^ of *S. enterica* was possible within 12 h including 8 h for cultivation.

## 1. Introduction

Rapid detection of food-borne pathogens is of great importance to public health and the food industry. Food-borne illnesses are often caused by bacteria, viruses, chemical agents or natural toxins. Half of all food-borne illnesses are caused by bacteria such as *Salmonella* spp., *Escherichia coli* or *Staphylococcus*. Especially, *Salmonella* *enterica* is the causative agent of human salmonellosis and one of the most notorious food-borne pathogens worldwide [1]. Infection by *S. enterica* is often caused by ingesting chicken meat or eggs. This is because chickens sometimes carry *S. enterica* in their intestines. Even if chickens or eggs are not infected by *S. enterica* when they are produced, after production of these materials, there are various opportunities for infection by *S. enterica*, before they are consumed, including during processing, transportation, packaging, sales, cooking and serving food. Therefore, rapid detection of *S. enterica* at all stages from production to eating is necessary to avoid an outbreak. There is a need for faster and simpler screening tests to detect *S. enterica* in not only foods but also food manufacturing plants.

The conventional method to detect *S. enterica* requires multiple subculture steps followed by biochemical tests. Although the conventional culture method is highly sensitive and can detect a few colony-forming units (CFUs)·mL^−1^ of *S. enterica*, it takes at least three days to confirm that food is not contaminated by any *Salmonella* species, and five to six days to clarify whether food is contaminated by *S. enterica* [2]. Separately, many researchers have reported the rapid detection of food-borne pathogens using the polymerase chain reaction (PCR) [3,4,5,6,7,8]. A number of PCR assays specific to different *Salmonella* genes such as the 16S rRNA gene [4], the *invA* gene [5], the *phoP* gene [6], the *phoE* gene [7] and the *fimA* gene [8] have been reported. The highly specific identification of pathogens is enabled by PCR in a few hours. However, Fachmann *et al.* reported that the limit of detection (LOD) of real-time PCR-based detection of *S. enterica* was 10^3^ CFUs·mL^−1^ [9], which is a higher concentration than that of the conventional culture method. In addition, it is necessary to reduce the amount of PCR reagents used to assay many samples because PCR reagents are expensive.

The microfluidic devices are useful to reduce the reagents because the handling of a small amount of reagent is enabled in the devices. Especially, the development of the centrifugally driven microfluidic platforms, which are generally referred to as “Lab-on-a-Disk”, is of great interest [10]. The advantage of Lab-on-a-disk is to eliminate bulky external pumps for liquid transfer. Sundberg *et al.* reported digital PCR on Lab-on-a-Disk in 2010 [11]. The PCR mixture that contained plasmid DNA was divided into one thousand 33 nL microchambers by spinning the disk. Then, the PCR mixture in the microchambers were separated by forcing mineral oil through the spiral channel and performed PCR in each microchambers. Schuler *et al.* also reported digital droplet PCR on Lab-on-a-disk in 2015 [12]. The PCR mixture contained DNA was divided into eleven thousands 1.8 nL droplets and PCR on the disk was performed. In these reports, they performed the PCR from not cells but only DNA.

We have developed a compact disc (CD)-shaped device for single cell isolation to detect isolated cells easily and rapidly without the need for a micro-pumping system [13]. By spinning the device, bacterial cells can be isolated in microchambers on the device, regardless of cell size. More specifically, when the concentration of bacterial cells is 200 cells·μL^−1^ or lower, almost all bacterial cells were isolated as a single cell into each microchambers on the device. Moreover, we successfully achieved sequential PCR of the *invA* gene of isolated *S. enterica* by hot cell-direct PCR without the need to extract DNA [14]. In this device, only 1 μL of PCR reagent is needed for one sample, so the amount of reagents consumed in our device is 25 times lower than that for conventional PCR, *i.e.*, 25 μL. Furthermore, in the conventional PCR method, 1 μL of PCR reagent in a tube is too little to perform PCR normally, because the reagent is evaporated. In our device, the LOD of *S. enterica* is 5 × 10^4^ cells·mL^−1^ [14]. Therefore, improvement of LOD is essential to detect *S. enterica* in food. Furthermore, since food may be contaminated by various bacteria, one objective of this study was to be able to detect *S. enterica* from other bacterial cells using the device. Therefore, at first, we examined the specific detection of *S. enterica* from a mixture of *S. enterica* and *E. coli*, which is the most common bacterium. The food sample selected for this study was ground chicken because *S. enterica* infection is often caused by consuming chicken. Moreover, we evaluated the speed of detection of *S. enterica* in food using a combination of culture and PCR to improve the LOD of *S. enterica* in the device. By combining the culture method and PCR on the CD-shaped device, our detection method was more rapid than the conventional culture method, and more sensitive than real-time PCR based method.

## 2. Experimental Section

### 2.1. Bacterial Cells

*S. enterica* and *E. coli* were purchased from RIKEN BRC (Tsukuba, Japan). Nutrient broth was purchased from Sanko Junyaku Co., Ltd. (Tokyo, Japan). Nutrient broth with 0.5% NaCl medium (NB medium) (pH 7.2) and buffered peptone water (BPW) (pH 7.2) was used to grow *S. enterica* while Lysogeny Broth (LB) medium (pH 7.0) was used as the growth medium for *E. coli*. Bacterial cells were cultivated in liquid media overnight at 37 °C. Cell concentration was confirmed under an optical microscope and diluted to the desired concentration with 10 mM phosphate buffer solution (PBS). Furthermore, the number of CFUs was confirmed by cultivation on agar medium at 37 °C for 24 h.

### 2.2. PCR Reagents and Real-Time PCR

PCR was performed using a CycleavePCR *Salmonella* Detection Kit Ver. 2.0 (TaKaRa, Tokyo, Japan). This kit contains a 2× Cycleave reaction mixture and a 5× SIN primer/probe mix. The Cycleave reaction mixture contains Taq polymerase, RNaseH, buffer, dNTP mixture and internal control DNA. The internal control DNA is a sequence non-related to the target gene and works to detect false negatives. When the target gene is not detected, and the internal control DNA shows a positive signal, this indicates the absence of PCR inhibition and that the concentration of the target gene in the sample is below the detection limit. When both target and internal control are not detected, this indicates that PCR does not occur properly and that a reaction inhibitory factor exists in the sample. The SIN primer/probe mix is a mixture of primer and probe to detect the *invA* gene and internal control DNA. The probe for the *invA* gene was labeled with 6-carboxyfluorescein (FAM) and a quencher. The probe for the internal control DNA was labeled with X-Rhodamine (ROX) and a quencher. The increase in fluorescence from FAM and ROX occurred after an amplification of the *invA* gene and internal control DNA, respectively. After the probe is hybridized to the PCR product, it is cut by RNaseH. Then fluorescence intensity is increased by uncoupling the quencher from the probe. The reaction mixture consisted of a 1× Cycleave reaction mixture, a 1× SIN primer/probe mix, 0.5 U·μL^−1^ Taq polymerase (TaKaRa EX Taq hot start version) and the desired concentration of the suspension of *S. enterica*. The reaction mixture without *S. enterica* was used as the negative control.

The number of washing treatments necessary to remove the inhibitor from ground chicken in PCR was also evaluated. In this experiment, 5 μL of *S. enterica* recovered by Percoll^®^ (GE Healthcare, Tokyo, Japan) was used as a template in real-time PCR, which was performed with a 7500 Real-Time PCR System (Applied Biosystems, Tokyo, Japan). Thermal cycling was initiated at 95 °C for 2 min to lyse *S. enterica*, followed by 40 cycles of 95 °C for 5 s, 55 °C for 10 s, and 72 °C for 30 s.

### 2.3. Fabrication of CD-Shaped Device

**Figure 1:**
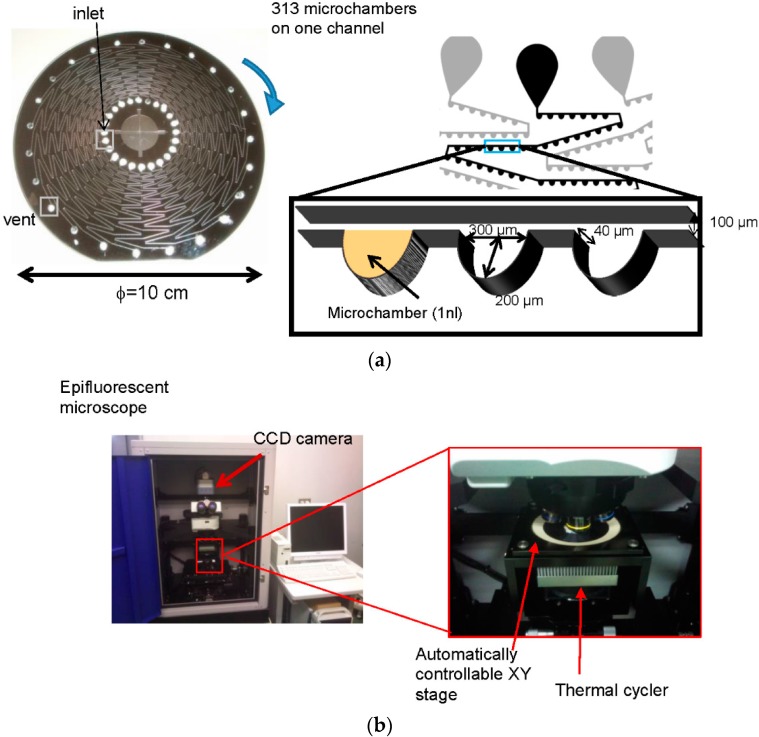
(**a**) design of the CD-shaped device; (**b**) the detection system was fabricated to acquire images of all microchambers automatically.

The CD-shaped device was fabricated according to a previously described method [14]. In brief, the microchannel and microchambers (40 μm in depth) were fabricated by deep reactive ion etching (deep-RIE) on a silicon wafer (ϕ = 10 cm; 525 μm thick). The etched silicon wafer was anodically bonded to a glass plate (500 μm thick) that has holes (ϕ = 2 mm) for inlets and vents bored by microblasting. As shown in Figure 1a, on a CD-shaped device, 24 zig-zag shaped microchannels are arranged, and 313 microchambers are arrayed on the outer side of each microchannel. The dimensions of the microchambers are 300 μm (width) × 200 μm (height) × 40 μm (depth), and the microchamber can accommodate approximately 1 nL of sample solution. The gap between microchambers is approximately 200 μm. To prevent the displacement of solution to neighboring microchambers, the surface of the microchannels and microchambers was modified by triethoxymethylsilane (Wako, Osaka, Japan). For this modification, we infused 3 μL of triethoxymethylsilane into each microchannel by capillary force then baked the device overnight at 80 °C. After baking, using a microscope, we confirmed that the triethoxymethylsilane did not remain in the microchannel and microchambers.

### 2.4. Detection of S. Enterica Using a CD Shaped Device

**Figure 2:**
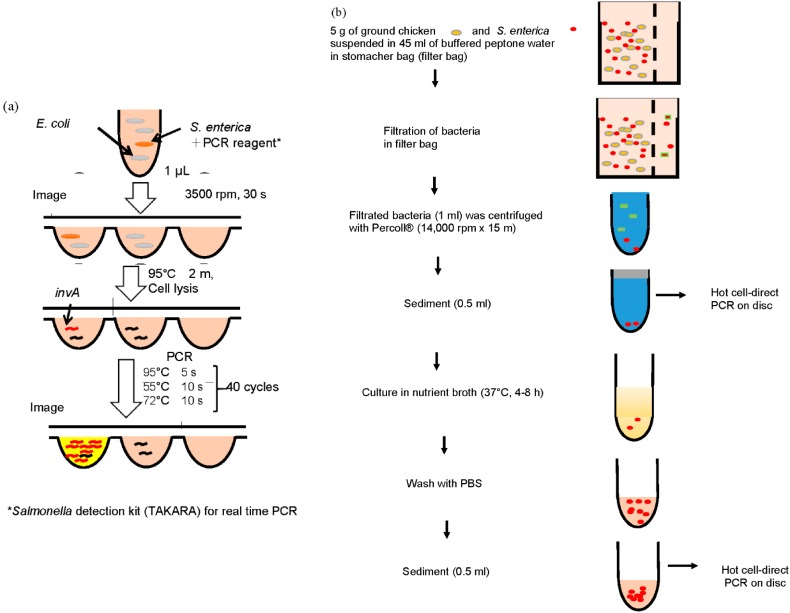
Detection scheme. (**a**) detection of *S. enterica* using the CD-shaped device by the detection system; (**b**) pre-treatment of chicken meat spiked with *S. enterica*.

We previously developed a detection system that could detect the fluorescence image of all microchambers in a microchannel of the CD-shaped device before and after PCR [15]. As shown in Figure 1b, the detection system is composed of an epifluorescence microscope and a charge coupled device (CCD) camera to detect microchambers, a thermal cycler for PCR, and a controllable XY stage for automatic detection of all microchambers in a microchannel. In this study, detection of *S. enterica* using the CD-shaped device was performed by the detection system as shown in Figure 2a. Briefly, 1 μL of reaction mixture was introduced into an inlet of the device. By spinning the device, the reaction mixture was completely isolated in the microchambers. Before PCR, the fluorescence intensity of each microchamber was measured by the detection system. Then, the CD-shaped device was placed on the stage of a thermal cycler (Astec, Tokyo, Japan). This stage was shaped to fit the CD-shaped device. Thermal cycling was initiated at 95 °C for 2 min as heat treatment to lyse *S. enterica*, followed by 40 cycles of 95 °C for 5 s, 55 °C for 10 s, and 72 °C for 10 s. After PCR, the fluorescence intensity of each microchamber was measured by the detection system. The relative fluorescence intensity (RFI) is expressed as:

(1) $$\mathrm{RFI}=\frac{\text{Fluorescence intensity after PCR}}{\text{Fluorescence intensity before PCR}}$$

As shown in Figure 2b, in the sample case with a low concentration of *S. enterica* in ground chicken, 500 μL of sediment after Percoll^®^ (GE Healthcare Japan, Tokyo, Japantreatment was mixed with 5 mL of Nutrient broth with 0.5% NaCl (NB) liquid medium and cultured at 37 °C for 4 or 8 h. After cultivation and washing with PBS, this *S. enterica* sample was detected on the CD-shaped device.

### 2.5. Separation of S. Enterica from Chicken

Bacterial samples from chicken meat as the food sample were prepared as follows. Ground chicken was obtained from a local grocery and used as the food sample. To recover bacteria from chicken meat, 5 g of ground chicken and 45 mL of BPW were mixed in a stomacher bag (Atect, Osaka, Japan). After stirring for 1 min, 100 μL of filtrate, which was passed through the filter of the stomacher bag, was cultivated on NB agar medium and LB medium at 37 °C to detect the existence of *S. enterica* and *E. coli*.

Samples were prepared for PCR as follows (Figure 2b). The desired concentration of *S. enterica* cells was spiked to 5 g of ground chicken and mixed with 45 mL of BPW in the stomacher bag. After stirring for 1 min, 1 mL of filtrate was mixed with 0.7 mL of Percoll^®^. After the mixture was centrifuged at 14000 rpm for 15 min at 4 °C, 0.5 mL of sediment was collected as the *S. enterica* sample. After washing the sample with 10 mM PBS one to three times, part of it served for hot cell-direct PCR while another part was cultivated on NB agar medium at 37 °C.

## 3. Results and Discussion

### 3.1. Detection of S. Enterica in Reaction Mixture with E. coli on CD-Shaped Device

**Figure 3:**
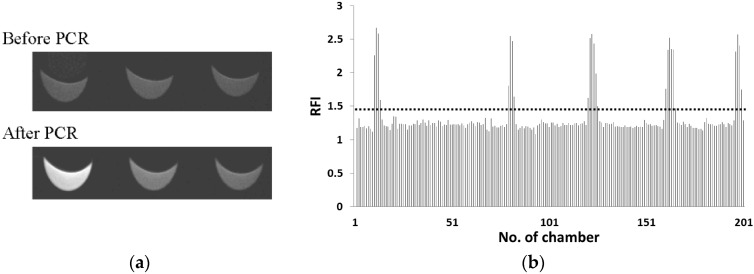
(**a**) fluorescence image of microchambers on the CD-shaped device before and after PCR; (**b**) RFIs of 200 microchambers from upstream to downstream for 50 cells·μL^−1^ of *S. enterica* with 1000 cells·μL^−1^ of *E. coli*.

The detection of *S. enterica* contamination in food would require discrimination from among various bacteria. Therefore, we examined whether *S. enterica* was selectively detected by our detection system from a sample containing many general bacteria. In this study, 10^3^ CFUs·μL^−1^ of *E. coli* was used as the general bacteria, because the standard value of the number of the general bacteria for non-heated food was less than 10^6^ CFUs·g^−1^. In this experiment, 50, 100 and 400 cells·μL^−1^ of *S. enterica* with 1000 cells·μL^−1^ of *E. coli* were examined. Figure 3a displays fluorescence images of the microchambers before and after PCR. RFI indicates the ratio of fluorescence intensity after PCR to that before PCR in this study. In this case, although the fluorescence intensity of the microchambers in the center and on the right did not increase, those on the left side increased clearly after PCR. Figure 3b shows the RFIs of 200 microchambers from upstream to downstream for 50 cells·μL^−1^ of *S. enterica* with 1000 cells·μL^−1^ of *E. coli*. To determine the detection threshold value of *S. enterica*, RFI of the negative control was measured on the CD-shaped device and was lower than 1.4. Therefore, microchambers displaying an RFI value greater than 1.4 were identified as those containing *S. enterica* in this study. The chamber with RFI value greater than 1.4 was observed around the turn of microchannel. We observe such localization on the isolation of rod-like bacteria such as *salmonella* sp. but no localization of ball-shaped cell and particle was observed [15]. Such localization might be caused by the shape of the isolated bacteria. As shown in Table 1, the number of microchambers with an RFI value greater than 1.4 was 19, 36, and 92 in 50, 100 and 400 cells·μL^−1^ of *S. enterica*, respectively. Furthermore, these numbers depended on the concentration of *S. enterica*. Therefore, we could selectively detect *S. enterica* in a sample containing large number of *E. coli* cells. In our previous report [14], the number of microchambers with high fluorescence intensity in 400 cells·μL^−1^ of *S. enterica* in our CD-shaped device was 90 microchambers among 200 microchambers. The observed number of microchambers with RFI value greater than 1.4 was larger than expected number. In this experiment, cell concentration was confirmed under an optical microscope but not by colony formation, so that we might detect dead *S. enterica* cells as well as living cells.

**Table 1:** Number of microchambers with relative fluorescence intensity (RFI) > 1.4.

Concentration of *S. enterica* (cells·μL^−1^)	Number of Microchambers with RFI > 1.4
50	19
100	36
400	92

### 3.2. Separation of S. enterica from Chicken

Generally speaking, ground chicken, which is purchased from a grocery store, has a small amount of bacteria. Therefore, before examining the recovery rate for *S. enterica*-spiked chicken, the number of bacteria contaminated in chicken purchased from a grocery store was confirmed by the colony counting method. The concentration of general bacteria contaminated in chicken was 200 CFUs·g^−1^, but no *S. enterica* cells were found. Generally, the infection by *S. enterica* is often caused by ingesting food contaminated by more than 10^5^ to 10^6^ CFUs [16]. Then, the recovery rate for *S. enterica*-spiked chicken was examined. At first, 2.3 × 10^7^ CFUs of *S. enterica* was added to 5 g of ground chicken and 45 mL of BPW in a stomacher bag. After treatment with Percoll^®^, the number of *S. enterica* cells, which were collected from *S. enterica*-spiked chicken, was confirmed by colony counting. The concentration of *S. enterica* was 2.7 × 10^6^ CFUs·g^−1^. Therefore, the recovery rate for *S. enterica* was 59% after Percoll^®^ treatment. The remaining 41% of *S. enterica* would likely be adsorbed to the surface of the ground chicken or the stomacher bag.

**Figure 4:**
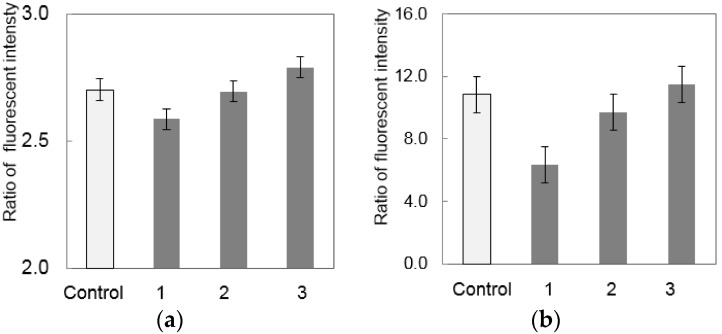
Evaluation of the efficiency of PCR for the number of washes. (**a**) RFI of FAM for the *invA* gene; (**b**) RFI of ROX for the internal control DNA. Error bars indicate standard deviation (SD) (*n* = 3).

To sufficiently remove the inhibitor to PCR in the food sample, the number of washes after Percoll^®^ treatment was examined. In this experiment, 1.5 × 10^9^ CFUs·g^−1^ of *S. enterica* was spiked to ground chicken. As a control sample, the same concentration of *S. enterica* in PBS was examined. As shown in Figure 4, when *S. enterica* was not spiked to chicken (control), the RFI of FAM for the *invA* gene and the RFI of ROX for the internal control DNA were 2.7 and 10, respectively. After Percoll^®^ treatment and washing one to three times, the RFI of FAM was 2.6, 2.7 and 2.8, respectively while the RFI of ROX was 6.3, 9.8 and 11, respectively. The low RFI of ROX observed after washing once indicated that the inhibitor in the food sample was not sufficiently removed. In contrast, the inhibitor in food samples was sufficiently removed when samples were washed at least twice. Therefore, the number of washes was set to two to sufficiently remove the inhibitor.

### 3.3. Detection of S. enterica from Chicken on CD-Shaped Device

After the number of washes was optimized, the *S. enterica* sample spiked to ground chicken was examined on the CD-shaped device. As the threshold value of RFI for the detection of *S. enterica* was 1.4 on the device, a microchamber with an RFI value greater than 1.4 was identified as one containing *S. enterica*. Spiked concentrations of 4.6 × 10^7^, 4.6 × 10^6^, 4.6 × 10^5^ and 4.6 × 10^4^ CFUs·g^−1^ were examined immediately after washing. As shown in Table 2, in the case of 4.6 × 10^7^, 4.6 × 10^6^, 4.6 × 10^5^ and 4.6 × 10^4^ CFUs·g^−1^, 170, 24, 6 and 0 microchambers, respectively exceeded the threshold. Furthermore, for every concentration, microchambers with an RFI value lower than the threshold were observed: the average RFI of those microchambers was approximately 1.2, almost equal to that of the negative control. In the case of 4.6 × 10^5^ CFUs·g^−1^, the concentration of *S. enterica* in the reaction mixture was expected to be 9 CFUs·μL^−1^. Taking the recovery into consideration, the number of microchambers that exceeded the threshold was expected to be three or four, while the observed number was six in this experiment. This result might suggest that dead *S. enterica* were detected by PCR. Furthermore, cultivation before PCR is necessary to detect *S. enterica* at concentrations lower than 4.6 × 10^4^ CFUs·g^−1^.

**Table 2:** Number of microchambers exceeded the threshold.

Concentration of *S. enterica* (CFUs·g^−1^)	Number of Microchambers Exceeded the Threshold
4.6 × 10^4^	0
4.6 × 10^5^	6
4.6 × 10^6^	24
4.6 × 10^7^	170

**Figure 5:**
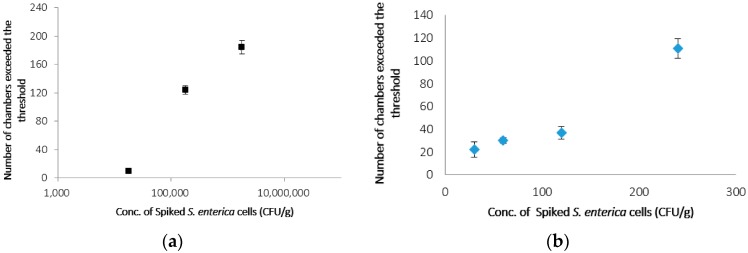
(**a**) number of microchambers exceeded the threshold after cultivation for 4 h; (**b**) number of microchambers exceeded the threshold after cultivation for 8 h. Error bars indicate standard deviation (SD) (*n* = 3).

To achieve highly sensitive detection, *S. enterica* collected from ground chicken following filtration and Percoll^®^ treatment was cultivated before PCR and detection on the CD-shaped device. Figure 5a indicates the number of microchambers that exceeded the threshold after PCR following cultivation for 4 h. The number of microchambers with 1.7 × 10^6^, 1.7 × 10^5^ and 1.7 × 10^4^ CFUs·g^−1^ was 184, 124 and 10, respectively. In contrast, without cultivation, six microchambers exceeded the threshold when *S. enterica* concentration was 1.7 × 10^6^ CFUs·g^−1^. Furthermore, when *S. enterica* concentration was 1.7 × 10^5^ and 1.7 × 10^4^ CFUs·g^−1^, no microchambers exceeded the threshold. Therefore, the detection of *S. enterica* by PCR when *S. enterica* concentration was 1.7 × 10^4^ CFUs·g^−1^ was confirmed within 8 h between sampling and detection. The number of microchambers that exceeded the threshold after cultivation for 8 h is shown in Figure 5b. In the case of 240, 120, 60 and 30 CFUs·g^−1^, the number of microchambers that exceeded the threshold was 105, 37, 30 and 20, respectively. The sensitive detection of 30 CFUs·g^−1^ of *S. enterica* was possible on the CD-shaped device in 12 h, which included 8 h of cultivation. These results demonstrated that this method is more than 300 times more sensitive than the real-time PCR method and more than 10 times as rapid as the conventional culture method. Furthermore, detection of the number of only viable bacterial cells is also important in food-borne illness. By cultivating *S. enterica* before PCR in the CD-shaped device, we could detect only viable *S. enterica* in a sample carrying a low concentration of this bacterium because not only viable *S. enterica* but also dead bacterial cells were not detected at less than 4.6 × 10^4^ CFUs·g^−1^ of *S. enterica* without cultivation.

## 4. Conclusions

The recovery rate for *S. enterica* after sample preparation was 59%. The inhibitor in the food sample was sufficiently removed after two washes with 10 mM PBS. The detection of *S. enterica* at 4.6 × 10^6^ CFUs·g^−1^ was confirmed after 4 h without cultivation before PCR. After cultivation for 4 h, *S. enterica* at 1.7 × 10^4^ CFUs·g^−1^ was detected after 8 h. Furthermore, the sensitive detection of 30 CFUs·g^−1^ of *S. enterica* was possible on the CD-shaped device within 12 h, which included 8 h for cultivation. Our detection method is more than 10 times as rapid as the conventional culture method and more than 300 times more sensitive than the real-time PCR method. Cultivating *S. enterica* before PCR allows for the rapid and sensitive detection of viable *S. enterica* in a sample of *S. enterica* in food.
